# Whole genome sequence data of *Lactobacillus fermentum* HFD1, the producer of antibacterial peptides

**DOI:** 10.1016/j.dib.2020.106105

**Published:** 2020-08-01

**Authors:** G.D. Ozhegov, A.S. Pavlova, D.E. Zhuravleva, N.E. Gogoleva, E.I. Shagimardanova, M.I. Markelova, D.R. Yarullina, A.R. Kayumov

**Affiliations:** a Kazan Federal University, Kazan, Russia; b Friedrich–Alexander University Erlangen, Nürnberg, Germany; c Kazan Institute of Biochemistry and Biophysics, Kazan Science Centre, Russian Academy of Sciences, Kazan, Russia

**Keywords:** *Lactobacillus fermentum*, Antibacterial peptides, De novo genome assembly, Illumina Miseq, Oxford nanopore

## Abstract

Here we report the whole genome sequence of *Lactobacillus fermentum* HFD1 strain, the producer of antibacterial peptides. The genome consists of one circular chromosome with 2101878 bp in length and GC-content of 51.8%, and includes linear DNA with 5386 bp in length with 100% identity to bacteriophage phiX174. The analysis of the genome has revealed 2049 genes encoding for proteins including 867 proteins without known function and 70 genes encoding for RNAs (10 rRNAs, 59 tRNAs and 1 tmRNA). Putative genes responsible for the biosynthesis of 4 antimicrobial peptides were identified. The NCBI Bioproject has been deposited at NCBI under the accession number PRJNA615901 (https://www.ncbi.nlm.nih.gov/bioproject/PRJNA615901/) and consist of full annotated genome and raw sequence data.

**Specifications Table**

**Table:** 

Subject	Biochemistry, Genetics and Molecular Biology (General)
Specific subject area	Genomics, microbiology, antibacterial peptides
Type of data	Table, figure, text files
How data were acquired	Illumina MiSeq; Oxford Nanopore MinIon; Brucker Biotyper; Unicycler; ADAM; AMPA; CAMPr3
Data format	Analyzed
Parameters for data collection	DNA were extracted by phenol-chloroform method and sequenced by ONT MinIon and Illumina MiSeq. Assembly were made by Unicycler asssembler.
Experimental factors	Strain HFD1 has been isolated from faeces of healthy woman and choosen during screening for antagonistic activity. Bacterial genomic DNA was extracted and sequenced by ONT MinIon and Illumina MiSeq. Sequenced genome was used for assembly, annotation and search of genes of antimicrobial compounds production.
Description of data collection	Whole circular genome
Data source location	Institution: Kazan Federal University City/Town/Region: Kazan, Republic of Tatarstan Country: Russian Federation
Data accessibility	Repository name: NCBI Bioproject Data identification number: PRJNA615901 Direct URL to data: https://www.ncbi.nlm.nih.gov/bioproject/?term=fermentum+hfd1
Related research article	Pavlova, A.S., Ozhegov, G.D., Arapidi, G.P., Butenko, I.O., Fomin, E.S., Alemasov, N.A., Afonnikov, D.A., Yarullina, D.R., Ivanov, V.T., Govorun, V.M. and Kayumov, A.R., Identification of Antimicrobial Peptides from Novel Lactobacillus fermentum Strain. The Protein Journal, 2020 Feb;39(1):73-84. doi: 10.1007/s10930-019-09879-8.

## Value of the data

• Data can be used for unraveling molecular mechanisms of probiotic activity of healthy human gut microbiota.
• Whole genome sequence data may be used for safety evaluation of *L. fermentum* HFD1 as probiotic or starter culture in the functional dairy food industry.
• The data provide a genetic basis of genotypic and phenotypic diversity of *L. fermentum*.

## 1 Data description

Lactobacilli were isolated from the feces of healthy woman. From 40 randomly selected colonies, an isolate producing antibacterial compounds capable of suppressing the growth of *P. aeruginosa* and *S. marcescens* was chosen [1]. Initial identification of the bacterial isolate by MALDI-TOF mass spectrometry on Brucker Biotyper and 16S rRNA sequencing revealed that the studied isolate is a representive of the species *Lactobacillus fermentum* (score 2.007 according to Brucker Biotyper and 99.9% identity with 16S rRNA gene of *L. fermentum* strain YL-11*,* GenBank: CP034193.1). The genome sequence of *L. fermentum* HFD1 on Illumina MiSeq platform was completed on April 2019 and sequence on ONT MinIon platform was completed on December 2019 and has been deposited to NCBI as Bioproject number PRJNA615901 as whole-genome contig and raw sequence data. Illumina sequencing yielded 5 million filtered pair-end reads obtaining 1.2 Gbp data. MinION generated 530 thousand reads with cumulative length of 1Gbp and maximum read length of 53364 bp. For *de novo* assembly of *L. fermentum* HFD1 genome from hybrid reads the best result was obtained when using Unicycler software [2], the short-read based assemblier with circulirization by long reads. While Spades [3] gave circular contig with close length, the Unicycler was chosen due to it built-in polishing steps. Other assemblers such as Canu [4] with Pilon [5] polishing or Flye [6] with Pilon [5] gave linear contigs ranging from 2.2 Mbp to 2.5Mbp in length. Even by using Circlator [7], special tool for contigs circulisation, circular contigs could not be obtained, probably due to overlaps between start and end of the contig. The final assembly allowed obtaining 2 circular contigs corresponding to *L. fermentum* circular chromosome with 2101878 bp in length and GC-content of 51.8% and bacteriophage phiX174 5386 bp in length and GC-content of 44%. The raw reads alignment with BWA [8] and post-assembly assessment showed that 99.9% of Illumina reads and 99.2% of MinION reads were mapped to assembled genome with error rates of 8 × 10^−4^ for Illumina and 0.1 for MinION. Finally, the genome was read with 575 × coverage for Illumina data and 454 × coverage for MinION data. Genome was annotated by PROKKA software [9] and contained 2112 total genes including 2042 CDS, 10 rRNAs and 60 tRNA. 867 genes encoded proteins without known biological function and identified as “hypothetical” or “putative”. With using RAST web-service [10] we established that 542 coding sequences encode proteins with known or predicted functions which can be distributed into 23 general metabolic groups (Table 1).

**Table 1:** The genes distribution within 23 general COG functional categories

Number of genes	Description
133	Amino Acids and Derivatives
132	Carbohydrates
119	Protein Metabolism (folding, biosynthesis, processing and modification, degradation)
91	Cofactors, Vitamins, Prosthetic Groups, Pigments (biosynthesis, degradation, metabolism)
85	Nucleosides and Nucleotides (conversion, synthesis, utilization)
47	DNA Metabolism (repair, RMS, replication, recombination)
43	Fatty Acids, Lipids, and Isoprenoids
24	Cell Wall and Capsule
35	RNA Metabolism
23	Virulence, Disease and Defense (resistance to antibiotics, toxic compounds and intracellular resistance)
4	Phosphorus Metabolism
14	Respiration
13	Stress Response
15	Miscellaneous
9	Membrane Transport
4	Regulation and Cell signaling
3	Potassium metabolism
13	Nitrogen Metabolism
5	Dormancy and Sporulation
8	Iron acquisition and metabolism
4	Cell Division and Cell Cycle
3	Sulfur Metabolism
6	Phages, Prophages, Transposable elements, Plasmids
1500	Other genes not included in COGs

*L. fermentum* HFD1 produces antibacterial compounds of peptide nature [1]. The analysis of ORFs encoding for putative small peptides and proteins with unknown function by using AMPA, ADAM and CAMPR3 algorithms [11,12,13] in order to predict their potential antimicrobial activities allowed revealing of 4 proteins as putative antimicrobial peptides (Table 2). The first of them (peptide 1) was found earlier in the fraction of *L. fermentum* HFD1 peptidome exhibiting antimicrobial activity and thus appears the most probable candidate as antimicrobial peptide.

**Table 2:** Peptides from *L. fermentum* HFD1 with predicted antimicrobial activity

№	The protein ID	Peptide sequence	ADAM	CAMPR3			AMPA		Length in peptidome
				SVM	Random Forest	DAC		Length	
1	QIX58482.1	MGLIWSLIVGAIIGAIAGAITNRGAAMGWIANIVAGLIGAWIGQGLLGTWGPSLAGMALIPSIIGAIILVLIVSLVVGRTGKK	1.52	1.0	0.566	0.527	0.24	84	14
2	QIX58771.1	MLSLITNTVIILLIIGCAFWQLVKVFKRAKKGKCAACDYDCAVKQQVLKQEKHGAQ	0.82	0.889	0.6715	0.81	0.2226	56	N/A
3	QIX58909.1	MAVKIKTPAGMIDIANDVIATVVGGAATDNYGVVGMASRNPLKDGVNQILGRDSFHQGVVIRQQDNGIAVDVYIIVGYGTKISAVSKSVQEKVKYNLEAMLGVTANSVNIMVQGVRVLGD	0.09	1.00	0.9855	1.0	0.248	120	N/A
4	QIX58994.1	MNNQSNVTPQNGNLQYKFCQNCGAKIDVKAVVCPKCGVPVNGNNAESNSEDRNNGWWNLLGFFFPIVGWILWAVWHKEYPKRAHGICVWSWVSFGISFVIGFVNGFLSTL	1.32	1.00	0.7745	1.0	0.237	110	N/A

## 2 Experimental design, materials, and methods

### 2.1 Isolation of lactobacilli and growth conditions

The *L. fermentum* strain HFD1 was isolated from the stool sample of a 27-year-old healthy woman in February 2015. The volunteer provided her written informed consent to participate in this study. The stool sample was self-collected and transported frozen to the laboratory. Upon receipt at the laboratory, five grams of feces were suspended in 45 ml of de Man, Rogosa, Sharpe broth (MRS broth, HiMedia, India) and pre-incubated under anaerobic conditions (Anaerogas gaspack, NIKI MLT, Russia) at 37 °C for 24 h. The resulting enrichment culture was serially 10-fold diluted in sterile phosphate-buffered saline (PBS) and plated onto MRS agar (HiMedia, India) followed with incubation under anaerobic conditions at 37 °C for 48 h. The isolate designed as HFD1 was preliminary assigned to the genus *Lactobacillus* since it was Gram-positive, catalase-negative, non-motile, non-spore forming, and exhibited typical morphology. Cells appeared as straight rods (0.5 μm × 2–4 μm) occurring singly and in pairs. Colonies on MRS agar were small (approximately 1 mm in diameter), round, convex, smooth, with entire margins, and white. Identification of the strain was completed by MALDI-TOF mass spectrometry (MALDI Biotyper system, Bruker Daltonik, Germany) [1] and genotyping. For further cultivation of the isolate, MRS broth (HiMedia, India) was used. Bacteria were grown at 37 °C under anaerobic conditions (Anaerogas gaspack, NIKI MLT, Russia).

### 2.2 Identification of HFD1 isolate

The identification of the HFD1 isolate was performed by MALDI-TOF mass spectrometry (Bruker Biotyper system, Bruker Daltonics, Germany) and genotyping [14,15]. Accordingly to the manufacturer recommendation, the spectrum of peaks of the unknown organism is compared to reference peak lists of organisms in the reference library and a log (score) value between 0.00 and 3.00 is calculated. The score of 2.00–3.00 corresponds to the high confidence identification, 1.70–1.99—low confidence identification, < 1.70—no organism identification. For the genotyping, the genomic DNA of isolate was purified by using diaGene bacterial DNA extraction kit (Dia-M, Russia) from 10 ml of 24 h old culture grown under static anaerobic conditions. Then the gene of 16S rRNA was amplified by using universal 16S rRNA bacterial primers 27F (5′-GAG TTT GAT CCT GGC TCA G-3′) and 1392R (5′ ACG TT CC TG TA GA TT-3′) and Q5 DNA polymerase and sequenced (Evrogen JSC, Moscow) [15], [16], [17], [18]. Species were identified on the basis of 16S rRNA gene sequences similarity obtained by its alignment with NCBI database using BLAST algorithm (https://www.ncbi.nlm.nih.gov/BLAST).

### 2.3 Genomic DNA preparation

15 ml of overnight culture was centrifuged at 5000g for 10 min at 4 ˚C and cells were resuspended in MilliQ water with lysozyme in concentration of 50 µg/ml and stored for 12 h at 37 °C with shaking at 200 rpm [19]. Then the genomic DNA was extracted by a phenol-chloroform approach with subsequent wash 3 times with chloroform. DNA quality was assessed by gel electrophoresis in 0.7% agarose gel and NanoDrop 2000.

### 2.4 DNA sequencing

For Illumina MiSeq sequencing DNA was sheared to fragments ranging between 300 and 500 bp using the Covaris S220. The fragmented DNA sample was end-paired, dA-tailed, and ligated to multiple adapters. The ligated products were purified and further enriched using PCR, and paired-end sequencing was performed by using Illumina Miseq (Illumina, USA).

The Native barcoding Kit (EXP-NBD104) and Ligation Sequencing Kit (SQK-LSK109) were used to prepare Nanopore sequensing library from total DNA without shearing. Library was sequenced on a MinION device using flow cell FLO-MIN106D R 9.4.1 Version (Oxford Nanopore Technologies). The basecolling and demultiplexing of reads has been performed by using MinKNOW software (version 19.12.5).

### 2.5 Genome assembly and quality check

For short-read based assembly with long-read circulation the Unicycler v.0.4.8 [2] was run in conservative mode with using spades v. 3.14.0 [3] with polishing by racon v. 1.4.3 and 8-step pilon (v. 1.23) polishing. Reads were aligned with BWA [8]. Canu v.1.8 [4] was run with all steps and contigs were circulated by Circlator v. 1.5.5 [7] and polished with short read by pilon v. 1.23 [5]. Flye v. 2.7 [6] was run in standard mode and contigs were polished with short read by pilon v. 1.23 [5].

### 2.6 Genome annotation

The genome has been annotated by using Prokka v. 1.14.6 software [9] with HMMER v. 3.2.1 and Barrnap for rRNAs and Rast web-server. Antibacterial peptides were predicted by using AMPA (http://tcoffee.crg.cat/apps/ampa/do) [11], ADAM (http://bioinformatics.cs.ntou.edu.tw/ADAM/) [12], CAMPR3 (http://www.camp3.bicnirrh.res.in/) [13]. The following thresholds were selected for positive decision regarding putative antimicrobial activity: >0.2 in AMPA, >0 in ADAM and >0.5 in CAMPR3 [1].

## Declaration of Competing Interest

Authors declare no conflicts of interests.

## Appendix Supplementary materials

Image, application 1
